# Genomic and ancestral variations linked to the development of post-acute sequelae of SARS-CoV-2 infection in Indian populations

**DOI:** 10.3389/fgene.2025.1696764

**Published:** 2026-01-06

**Authors:** Pooja Umesh Shenoy, Hrushikesh Udupa, A. I. Ananthakrishnan, Punya Sunil, Urvinder Kaur Sardarni, Narendra Kumar, Arpan Acharya, Siddappa N. Byrareddy, Priyanka Upadhyai, Ranajit Das

**Affiliations:** 1 Division of Data Analytics, Bioinformatics and Structural Biology, Yenepoya Research Centre, Yenepoya (Deemed to be University), Mangalore, Karnataka, India; 2 Department of Community Medicine, Yenepoya (Deemed to be University), Mangalore, Karnataka, India; 3 Department of Pharmacology and Experimental Neuroscience, Durham Research Center 8052, 985800 Nebraska Medical Center, Omaha, NE, United States; 4 Department of Medical Genetics, Kasturba Medical College, Manipal Academy of Higher Education, Manipal, India

**Keywords:** post-acute sequelae of COVID-19, COVID-19 host-genetics, genome-wide association study, genetic epidemiology, multiomics

## Abstract

**Background:**

Susceptibility to infectious diseases is a result of complex interactions between genomic, environmental, and clinical factors. COVID-19 severity and post-acute sequelae of COVID-19 (PASC) vary widely among individuals, yet its genetic determinants remain underexplored in Indian populations. In this article, we undertake an exploratory analysis to investigate candidate genetic variants and biological pathways underlying the clinical outcomes in COVID-19 severity and PASC.

**Methods:**

Sixty individuals with a history of COVID-19 were genotyped, and their data were supplemented with publicly available datasets from the Genome Asia 100K and Gujarat Biotechnology Research Centre. Two case–control genome-wide association study (GWAS) models were analyzed: (i) COVID-19 severity (mild/asymptomatic vs. severe) and (ii) an exploratory, hypothesis-generating GWAS for PASC (presence vs. absence of post-COVID-19 complications). Candidate genes identified here were further compared with RNA-sequencing datasets derived from brain and lung tissues of SARS-CoV-2-infected hamsters. The population-specific genetic risk for PASC was estimated using the polygenic risk score algorithm PRSice-2.

**Results:**

GWAS identified candidate genes common to both COVID-19 severity and PASC, including *CNTNAP2*, *WWOX*, and *ADAMTS17*, which are implicated in extracellular matrix remodeling and neurological and cognitive development. We identified 806 candidate genes shared between the severity and PASC cohorts. Of these, 30 protein-coding genes were associated with neuropsychiatric disorders, and 23 were linked to cardiovascular conditions. Notably, *CACNA1C*, *SLC8A1*, *GRK5*, *PDE4B*, and *LRRK2* were identified in both categories, suggesting potential convergence of molecular pathways underlying neurological and cardiovascular dysfunction. Integration with transcriptomic data reinforced the involvement of shared molecular pathways disrupted by SARS-CoV-2 infection. Polygenic risk analysis revealed significant population-specific variation in genetic predisposition to PASC.

**Conclusion:**

Genetic susceptibility to severe COVID-19 and PASC in Indian populations appears to be linked to dysregulation of pathways central to cardiac and neurological function. These findings, derived from an exploratory PASC GWAS, provide preliminary insights into the molecular mechanisms that may underlie the post-viral sequelae. These emphasize the need for population-wide genomic studies to validate the candidate associations, better understand PASC risk, and facilitate the development of precision diagnostics and therapeutics.

## Graphical Abstract

**Figure:**
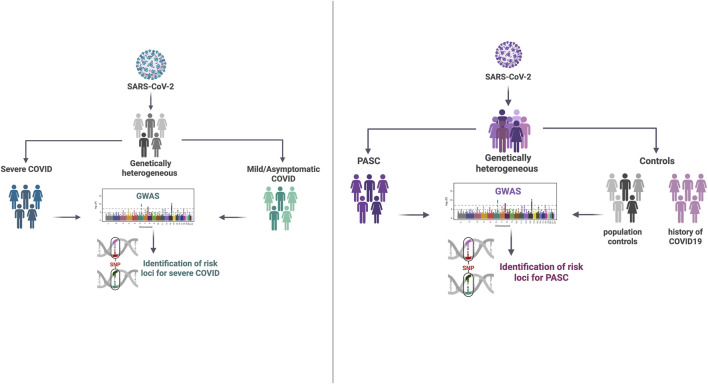

## Introduction

During infection, the severe acute respiratory syndrome coronavirus 2 (SARS-CoV-2) uses intricate mechanisms to exploit host-cell processes and evade antiviral defenses (Steiner et al., 2024), leading to a broad spectrum of manifestations ranging from asymptomatic to severe clinical outcomes (Gandhi et al., 2020; Rothan and Byrareddy, 2020). A series of genome- and exome-wide association studies using population-based or asymptomatic/mildly affected individuals as controls pointed to the heterogeneity of host genetic makeup as an important modulator of the clinical variability and severity of coronavirus disease 19 (COVID-19) infections (Ellinghaus et al., 2020; Li et al., 2021; Mousa et al., 2021; Niemi et al., 2021; Pairo-Castineira et al., 2021; Shelton et al., 2021; Upadhyai et al., 2021; Kousathanas et al., 2022; Upadhyai et al., 2022). The latest release and meta-analysis work by the Host Genetics Initiative, investigating both European and non-Caucasian ancestries across 35 countries, inferred single-nucleotide variants (SNVs) in several genes, including those previously reported to be linked with SARS-CoV-2 infection, critical illness, and hospitalization—for example, *solute carrier family 6 member 20 (SLC6A20)*, *alpha 1-3-N-acetylgalactosaminyltransferase and alpha 1-3-galactosyltransferase (ABO)*, *surfactant protein D (SFTPD)*, and *transmembrane serine protease 2 (TMPRSS2)* (viral entry); *mucin 1* (*MUC1)* and *mucin 5B* (*MUC5B)* (respiratory mucosal defense); *interferon alpha and beta receptor 2* (*IFNAR2) subunit*, *2'-5'-oligoadenylate synthetase 1* (*OAS1)*, and *tyrosine kinase 2* (*TYK2)* (type I interferon pathway); and forkhead box P4 (*FOXP4), SFTPD*, and *dipeptidyl peptidase 9* (*DPP9)* (lung homeostasis) (Kanai et al., 2023). Novel loci, such as mucin 4 (*MUC4)*, *mucin 16* (*MUC16*)*, Janus kinase 1 (JAK1), interferon regulatory factor 1 (IRF1)*, *interferon alpha 10* (*IFNA10)*, *calcium and integrin binding family member 4 (CIB4)*, *nephronectin (NPNT)*, *zinc finger with KRAB and SCAN domains 1 (ZKSCAN1), ATPase phospholipid transporting 11A (ATP11A),* and *proteasome 26S subunit*, *non-ATPase 3 (PSMD3)*, were also associated with variable COVID-19 morbidity.

Increasing evidence has revealed long-term clinical complications and multisystemic sequelae among COVID-19 patients who “recovered” from acute illness, termed as post-acute sequelae of COVID-19 (PASC) or “long COVID.” The World Health Organization (WHO) defines PASC as new or recurring health complications in individuals with probable or confirmed SARS-CoV-2 infection, usually 3 months after the onset of COVID-19, with symptoms that last at least for 2 months and cannot be explained by an alternative diagnosis (Author Anonymous, 2022). Estimates suggest that at least 65 million people could be affected by PASC worldwide (Ballering et al., 2022; Davis et al., 2023), with its prevalence likely ranging from 27% to 35% in the US (Statistics, 2022; Khan and Becker, 2024). The incidence of PASC was estimated at 10%–30%, 50%–70%, and 10%–12% among non-hospitalized, hospitalized, and vaccinated individuals, respectively (Al-Aly et al., 2022; Ayoubkhani et al., 2022; Ceban et al., 2022). A 3-year follow-up study showed that hospitalized patients faced a substantially higher risk of mortality and health loss (Cai et al., 2024).

PASC can involve mild to severe health complications affecting the cardiovascular, thrombotic, cerebrovascular, neurological, pulmonary, and endocrine systems and can manifest with over 200 different symptoms, including fatigue, dyspnea, and pulmonary dysfunction (Author Anonymous, 2022; Davis et al., 2023). Even patients without a history of comorbidities are highly liable to develop PASC, such as myocardial derangements (Dehghan et al., 2024) and new-onset type 2 diabetes in adults (Harding et al., 2023) and children (Miller et al., 2024). A UK-based epidemiological study noted that 7.2% of young individuals (11–17 years) showed a median of 5–6 symptoms of PASC at 3-, 6-, 12-, and 24-months post-infection, with repeated SARS-CoV-2 infection exacerbating its severity (Stephenson et al., 2024). New-onset neurocognitive and mood disorders, along with other neurological symptoms, including brain fog, headache, sensory dysregulation, balance issues, hearing loss, autonomic dysfunction, and stroke, have also been reported among individuals with PASC (Crivelli et al., 2022; Spudich and Nath, 2022).

Neuroimaging studies report that PASC can involve structural and functional brain abnormalities, such as a reduction in gray matter and brain volume (Douaud et al., 2022), decreased neurovascular perfusion in patients with chronic cognitive deficiencies (Ajčević et al., 2023), immune dysregulation in the cerebrospinal fluid (Mina et al., 2023), and a significant increase in neuroinflammation in many regions of the brain (VanElzakker et al., 2024). In some studies, the risk of cognitive derangements was found to be comparable among hospitalized and non-hospitalized COVID-19 patients (Woo et al., 2020).

The risk and severity of PASC can vary with age and the acuteness of viral infection (Huang et al., 2021; Han et al., 2022), yet cases also occur among asymptomatic or mildly affected COVID-19 patients (Malkova et al., 2021). Contributing factors for this include gender differences and preexisting comorbidities, such as obesity, diabetes, smoking, and socioeconomic status (Subramanian et al., 2022; Yu et al., 2024). Genetic predisposition to COVID-19 may further modulate susceptibility to PASC-related health complications, such as fatigue, headache, pneumonia and airway infections, and heart failure (Shenoy et al., 2023), but this remains largely unexplored outside European populations.

The Indian subcontinent is a region of remarkable diversity; its genetic makeup has been shaped by multiple waves of archaic gene flow (Reich et al., 2009; Basu et al., 2016; Narasimhan et al., 2019; Kumar et al., 2023; Kerdoncuff et al., 2024), along with stringent socioeconomic and cultural practices that have created over 5,000 anthropologically well-defined and largely endogamous populations, including >700 recognized as scheduled tribes (Mastana, 2014). It offers a unique opportunity to investigate population-specific genetic determinants of infectious disease response. Previous Indian studies have focused mainly on the evolution of the SARS-CoV-2 variants during successive waves of the COVID-19 pandemic (Misra et al., 2023; Ravi et al., 2023; Jena et al., 2024; Selvavinayagam et al., 2024; Wadhwa et al., 2024), with few reporting host–genetic associations related to COVID-19 severity—e.g., *rs1981458* in *furin*, *paired basic amino acid cleaving enzyme (FURIN)* (Pandey et al., 2024) and *rs479200* in *Egl-9 family hypoxia inducible factor 1* (*EGLN1)* (Harit et al., 2024); non-synonymous SNVs in *angiotensin converting enzyme 2 (ACE2)* that encodes for the receptor of the SARS-CoV-2 spike protein were predicted to alter individual susceptibility to SARS-CoV-2 omicron subvariants (Samanta et al., 2023).

The incidence and clinical spectrum of PASC in India remain poorly defined. One study suggested that elevated and persistent D-dimer levels may be associated with a high risk for PASC (Kalaivani and Dinakar, 2022). A retrospective observational study in the western Indian state of Maharashtra reported ∼6.48% of PASC cases, with ∼1.94% of patients developing new conditions, such as lung fibrosis, asthma, and hypertension (Karyakarte et al., 2023). Another work indicated that ∼64% of individuals experienced PASC symptoms with higher body mass index (BMI), obesity, hypertension, and abnormal chest X-ray as risk factors (Thyagaraj et al., 2022). A population-based study examined PASC symptoms over 6 months in the northern Indian state of Haryana and reported a significant persistence of cough among individuals with PASC, with higher risk observed in women (Chaudhry et al., 2024). However, none of these have assessed host genetic variations.

Given the lacunae in understanding the genetic variations associated with the spectrum of COVID-19 severity and the risk of PASC in Indian populations, we hypothesized that the genetic factors contributing to acute COVID-19 may also increase vulnerability to PASC, especially through pathways governing cardiovascular and neurological dysfunction. To address this gap, we conducted genome-wide genotyping of a cohort of 60 individuals with a history of SARS-CoV-2 infection in southern India using Infinium Global Screening Array v3 (GSA v3). These data were integrated with clinical and genomic datasets from 474 COVID-19 patients from the Gujarat Biotechnology Research Centre (GBRC), forming the largest Indian COVID-19 host-genome dataset to date. Leveraging this resource, we explored candidate genetic variants and molecular pathways associated with COVID-19 severity and risk of PASC among Indian populations.

## Methods

### Data acquisition

Saliva samples were collected from 60 individuals with a history of COVID-19 (*YenCOVID* dataset) at Yenepoya (deemed to be University), Mangalore, India. This study was approved by the institutional ethics committee (YEC-1/2021/034 and YEC-1/2023/236). Based on a self-reported questionnaire for clinical symptoms, these individuals were classified as asymptomatic, mild, moderate, and severe (Supplementary Table S1). The questionnaire covered COVID-19 symptoms, treatment regimens, PASC symptoms (if any), demographic details, personal medical and infection history, family medical history, and vaccination and travel details. Individuals were classified as PASC-positive if they reported ≥1 persistent symptom ≥3 months after acute infection onset, lasting ≥2 months without an alternative clinical explanation, in accordance with the WHO definition (Supplementary Table S2). Genomic DNA was extracted from the saliva samples and genotyped using the Infinium Global Screening Array-24 v3.0 BeadChip platform, which analyzed 648,465 SNVs. The *YenCOVID* data are reposited in the Science Data Bank (https://www.scidb.cn), with restricted access.

Genomic data and associated demographic and medical history details were obtained from the Gujarat Biotechnology Research Centre (GBRC). This dataset included genotyping data assessing 804,934 SNVs from 474 anonymized COVID-19 patients, who were genotyped using the Axiom Affymetrix genotyping array. It was merged with the *YenCOVID* dataset, and the combined dataset is known as *GBRC + YenCOVID*. It included 534 individuals and focused on 104,741 SNVs, which were common to both datasets. VCFtools v0.1.16 (Danecek et al., 2011) and PLINK v1.9 (Purcell et al., 2007) were utilized for all file conversions and data manipulations.

The symptoms reported by individuals in the *GBRC and YenCOVID* datasets included severe sore throat, dysphagia, fever, anosmia and ageusia, myalgia, headache, fatigue, nausea, cough, rhinorrhea, dyspnea, reduced appetite, respiratory distress, acute gastrointestinal symptoms, claustrophobia/anxiety, cognitive impairment, and cerebral thrombosis. Based on participants’ responses, we applied the criteria outlined in Supplementary Table S1 to categorize them into two groups: 317 were classified as asymptomatic/mild (controls), and 217 were classified as moderate/severe (cases).

**FIGURE 1:**
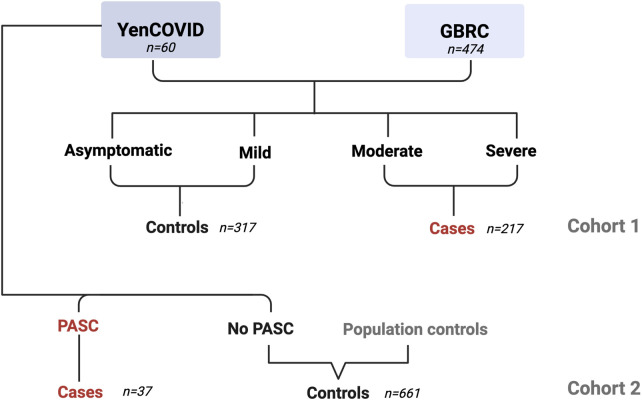
Schematic diagram of the distribution of participants recruited in the study.

The *YenCOVID* data were further supplemented with a control genomic dataset from Genome Asia 100K (*GaSP*) (100 Kconsortium, 2019), which included whole-genome data with 66,891,231 SNVs from 1,163 anonymized individuals of predominantly South and East Asian ancestry, including Indian populations, to generate a combined dataset (*GaSP + YenCOVID*) with 1,223 individuals. We focused on 553,102 SNVs that were common to both datasets (Figure 1).

### Genome-wide association study

We conducted a genome-wide association study (GWAS) in two different cohorts with distinct objectives. In the first cohort, using the *GBRC + YenCOVID* dataset, we aimed to identify genetic variants with significant frequency differences between moderate/severe and asymptomatic/mild COVID-19 patients (severity cohort). In the second cohort, we aimed to identify genetic variants with significant frequency differences between individuals with and without PASC using the *GaSP + YenCOVID* dataset. All individuals from the *GaSP* dataset were considered controls (PASC cohort). Standard case–control-based association analyses were performed in PLINK v1.9 using the --assoc command. In view of the modest sample size and exploratory nature of our population-specific analysis, we applied a suggestive significance threshold of p < 1 × 10^−4^ for primary variant selection (Hammond et al., 2021), consistent with prior COVID-19 GWAS frameworks that used relaxed thresholds for initial discovery (Ferreira et al., 2022; Lammi et al., 2025). Genome-wide significance thresholds were subsequently considered for replication-level prioritization. The −log_10_Ps of all assessed SNVs were plotted as Manhattan plots using the ‘qqman’ package in R v3.5.2. Significant SNVs were annotated using the SNVnexus (https://www.SNV-nexus.org/v4/, 12.12.2024) web-based server for GRCh38/hg38 (Oscanoa et al., 2020).

**FIGURE 2:**
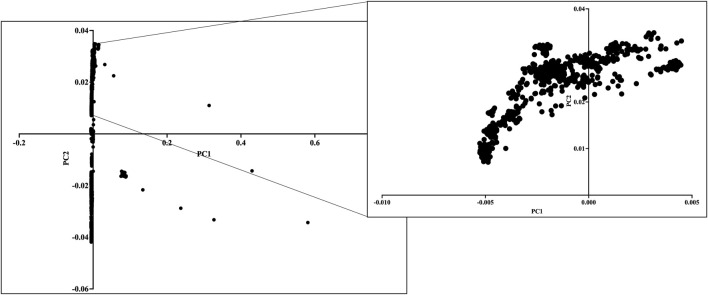
PCA of the GaSP + YenCOVID dataset. We restricted our downstream analysis to 698 South Asians (inset), comprised of individuals having post-acute sequelae of SARS-CoV-2 infection (PASC) (N = 37), individuals without PASC (N = 23), and population control from the GaSP dataset (N = 638). PC1 and PC2 explain major ancestral substructure; clustering indicates the subset selected for South Asian-specific analysis. We removed all non-South Asian samples in the dataset using PCA.

Before performing GWAS for the PASC cohort, the population structure was assessed within the *GaSP + YenCOVID* dataset using principal component analysis (PCA) implemented in PLINK v1.9 with the --pca command. The first two principal components (PC1 and PC2) were plotted in RStudio v1.4.1717 (Figure 2). To control for population stratification and avoid confounding due to genetic makeup, we restricted our analysis to 698 individuals of South Asian origin. Of the 698 individuals, those reporting PASC were treated as cases (N = 37), and the remaining were utilized as controls (N = 661).

### Transcriptomic analysis

Golden Syrian hamsters (GSHs) (*Mesocricetus auratus*), one of the small animal models, are widely used to study the disease pathogenesis of SARS-CoV-2 and its variants (Mohandas et al., 2021; Case et al., 2023; Furusawa et al., 2023; Kaur Sardarni et al., 2025; Rajaiah et al., 2024). To compare human data with animal models as described previously (Pandey et al., 2021; Poli et al., 2024; Rajaiah et al., 2024), hamsters (aged 6–8 weeks) were inoculated with the SARS-CoV-2 Omicron variant (Lineage B.1.1.529; Omicron Variant; # NR-56481 obtained from BEI Resources) and necropsied at week 5 post-infection to model PASC in humans (Frere et al., 2022). The number of samples used for transcriptomic analyses was as follows: young male lung, N = 3 for both controls and infected; young male brain, N = 3 for both controls and infected. The sample size was restricted to N = 3 per group to comply with ethical and biosafety restrictions on BSL-3 SARS-CoV-2 work. In our previous study, we demonstrated that GSHs showed differential immunometabolic responses to Omicron and delta variants of SARS-CoV-2 during acute infection (Rajaiah et al., 2024; Kaur Sardarni et al., 2025). Although genotyping data are not available, most infections in the clinical setup likely occurred during the Omicron-dominant wave in India, based on epidemiological timelines (Chavda et al., 2023; Triambak et al., 2023). Therefore, to model human disease pathogenesis and understand the molecular mechanisms underlying the development of PASC in our clinical cohort, we infected the GSHs with Omicron variants of SARS-CoV-2.

We performed the transcriptomic profiling of lung and brain tissues using RNA. One microgram of RNA per sample was used to prepare the rRNA-depleted cDNA library. The sequencing libraries were then generated and subjected to sequencing on an Illumina NovaSeq 6000 platform, generating 150 bp paired end reads. The adapter trimmed FASTQ files were aligned with the *Mesocricetus Auratus* reference genome (Mesocricetus_auratus.MesAur1.0.111. gtf) retrieved from Ensembl using STAR (v2.7.9a). The differential gene expression analysis of the SARS-CoV-2-infected GSH vs. uninfected controls was performed using the DESeq2 package in R (Love et al., 2014). The sequence data used in this study are available with NCBI SRA under BioProject number PRJNA1196444 (BioProject accession # PRJNA1222984).

### Population-specific variation in genetic predisposition toward PASC

Well-known polygenic risk score (PRS) estimation software, PRSice-2 (https://choishingwan.github.io/PRSice) (Choi and O'Reilly, 2019), was used to estimate population-specific PRS for PASC among individuals in the *GaSP* dataset. The GWAS summary statistics of PASC were obtained from an existing dataset (Lammi et al., 2025) that included 6,450 PASC cases and 1,093,995 population controls from 24 studies across 16 countries. This summary statistics file was used as the reference (--base) input for PRS computation. The effect sizes (beta values) and corresponding standard errors of the SNVs were extracted from the GWAS summary statistics file.

## Results

### Association of intrinsic and extrinsic risk factors with symptomatic COVID-19 and mortality

First, we utilized the demographic and medical history data from GBRC to examine the association between preexisting comorbidities and an increased risk of symptomatic COVID-19 and associated mortality (Table 1). Individuals with type 2 diabetes had an 8.8-fold increased risk of developing symptomatic COVID-19 (*p* < 0.0001). Those with heart disease faced a 14.7-fold higher risk (*p* < 0.0001), and people with hypertension had a 16-fold elevated risk (*p <* 0.0001) of developing symptomatic SARS-CoV-2 infection. Pulmonary disorders were associated with a 6.6-fold higher risk of symptomatic COVID-19 (*p <* 0.0001).

**TABLE 1:** Association between various demographic and other intrinsic and extrinsic risk factors with symptomatic COVID-19 and its mortality.

COVID-19 clinical outcomes	Risk factor	Odds ratio (95% CI)	Relative risk	*p*-value
Symptomatic COVID-19	Diabetes	8.837 (3.958–19.02)	1.389 (1.283–1.496)	<0.0001
	Heart disease	14.72 (4.113–61.87)	1.376 (1.266–1.471)	<0.0001
	Hypertension	15.96 (5.471–48.88)	1.416 (1.315–1.519)	<0.0001
	Pulmonary disorders	6.626 (2.166–20.57)	1.313 (1.170–1.412)	0.0001
	Age	4.291 (2.606–7.143)	3.427 (2.197–5.391)	<0.0001
COVID-19 mortality	Gender	0.6713 (0.4313–1.051)	0.7223 (0.5043–1.044)	0.092
	Diabetes	2.455 (1.502–3.977)	2.034 (1.411–2.888)	0.0004
	Heart disease	3.742 (2.163–6.449)	2.690 (1.848–3.801)	<0.0001
	Pulmonary disorders	2.965 (1.574–5.375)	2.274 (1.471–3.345)	0.0009
	Thyroid disease	2.473 (0.9215–6.504)	1.982 (0.9480–3.487)	0.104
	Fever	1.415 (0.9081–2.201)	1.333 (0.9286–1.919)	0.1233
	Cough	1.981 (1.244–3.200)	1.773 (1.192–2.661)	0.0048
	Dyspnea	4.373 (2.625–7.459)	3.494 (2.221–5.547)	<0.0001
	Chest pain	3.479 (1.617–7.295)	2.487 (1.479–3,797)	0.0022
	Nausea	2.505 (1.090–5.764)	2.004 (1.054–3.350)	0.0495
	Myalgia	1.450 (0.7550–2.897)	1.348 (0.7762–2.209)	0.3391

Age emerged as a significant risk factor for COVID-19 mortality, with older patients having a 4.3-fold increased risk of death (*p* < 0.0001) (Table 1). In contrast, gender was not a significant predictor of COVID-19-related mortality (*p =* 0.0920). Subjects with preexisting conditions, such as type 2 diabetes, faced a 2.4-fold higher risk (*p* = 0.0004), and those with heart disease had a 3.7-fold increased chance (*p* < 0.0001) of mortality. Pulmonary disorders were linked to a 3-fold increase in COVID-19-related mortality (*p* < 0.0009). Symptoms such as cough, dyspnea, chest pain, and nausea exacerbated the risk of mortality by approximately 2-fold (*p* < 0.0048), 4.4-fold (*p* < 0.0001), 3.5-fold (*p* < 0.0022), and 2.5-fold (*p* < 0.0495), respectively (Table 1).

Consistent with trends in global populations (Richardson et al., 2020; Dessie and Zewotir, 2021), old age and the presence of pre-existing comorbidities, such as type 2 diabetes, cardiovascular diseases, hypertension, and pulmonary complications, significantly exacerbated the risk of hospitalization and mortality among COVID-19 patients in India. However, unlike some studies (Nguyen et al., 2021), male gender was not significantly associated with adverse COVID-19 outcomes in our cohort.

These clinical and demographic findings formed the basis of further GWAS on two separate cohorts: (1) the first (*severity cohort*) utilized the *GBRC + YenCOVID* dataset (*see Methods*), comparing moderate/severe and asymptomatic/mild COVID-19 to identify genetic variants associated with COVID-19 severity; (2) the second (*PASC cohort*) utilized the *GaSP + YenCOVID* dataset (*see methods*), comparing individuals with and without PASC, and aimed to identify genetic variants that predispose to post-acute sequelae. Participants in the GaSP dataset were treated as controls for the PASC cohort.

### GWAS examining COVID-19 severity

**FIGURE 3:**
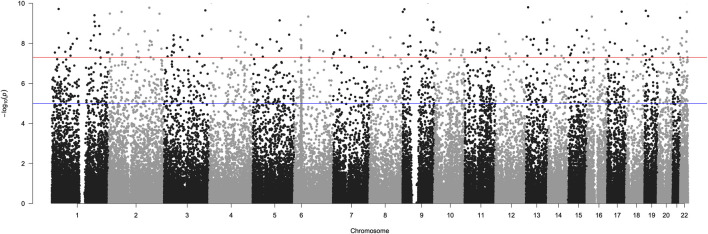
Manhattan plot summarizing GWAS results for the severity of COVID-19. The X-axis represents chromosomes (chr 1 to chr 22). SNVs present in the chromosomes are designated with dots. Negative log-transformed (−log_10_) multiple-testing-corrected *p*-values are plotted on the Y-axis. Genomes from 217 patients with moderate/severe symptoms were compared with those from 317 asymptomatic/mild patients. Of 1,04,741 SNVs used, 3,148 SNV markers revealed highly significant variation between asymptomatic/mild and moderate/severe cases. The SNVs with *p* < 0.00001 are indicated with the blue line, and those with *p* < 0.0000001 are indicated with the red line.

We examined the genomes of asymptomatic/mild COVID-19 patients (N = 317; controls) versus those that are moderate/severe (N = 217 cases) using the *GBRC + YenCOVID* dataset. Out of 1,04,741 SNVs used in genome-wide analysis, 3,148 autosomal SNVs across 2,116 genes showed significant association with the severity of COVID-19 (*p* < 0.0001) (Figure 3). SNVs showing highly significant association (*p* < 0.0001) mapped to pathways related to neuronal signaling, axon guidance, neural cell adhesion molecule interactions and acetylation, extracellular matrix (ECM) organization, olfactory signaling, Rho-GTPase cycle, and G- α signaling events (Supplementary Figure S1). Candidate genes harboring highly significant SNVs were enriched in GO terms (biological processes), such as membrane and cell adhesion, neurodevelopment, and neurogenesis (Supplementary Figure S2). In addition, significant SNVs were identified in genes associated with neuropsychiatric pathways.

### GWAS assessing PASC

**FIGURE 4:**
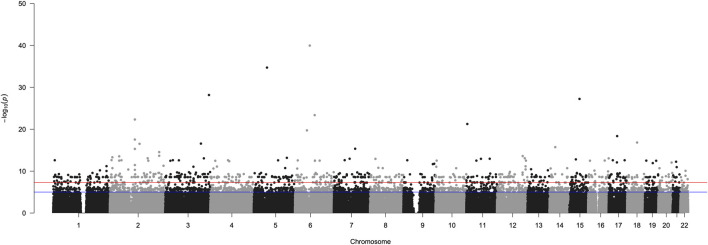
Manhattan plot summarizing GWAS results for the PASC. The X-axis represents chromosomes (chr 1–22). SNVs present in the chromosomes are designated with dots. Negative log-transformed (−log10) multiple-testing-corrected *p*-values are plotted on the Y-axis. A total of 37 COVID-19 patient genomes with PASC were compared with 661 without PASC, evaluating 5,53,102 SNVs. Among these, 8247 SNV markers revealed highly significant variation between individuals with and without PASC. The SNVs with *p* < 0.00001 are indicated with the blue line, and those with *p* < 0.0000001 are indicated with the red line.

We tested genomes of individuals of Asian ancestry from the GenomeAsia database (*N* = 661; controls) against those who reported PASC (*N* = 37; cases) using the *GaSP + YenCOVID* dataset, followed by PCA correction (see Methods). Of the 5,53,102 SNVs used in genome-wide analysis, 8,247 autosomal SNVs in 4,769 candidate genes showed significant association with PASC (*p* < 0.0001) (Figure 4).

SNVs showing highly significant association (*p* < 0.0001) were associated with pathways such as neuronal development, immune and cytokine signaling, olfactory transduction, and G-protein-coupled receptor (GPCR) signaling, along with infectious disease, ECM organization, and telomere maintenance (Supplementary Figure S3). Candidate genes harboring highly significant SNVs were enriched in GO terms (biological processes) such as neurodevelopment, neurogenesis, and neurodifferentiation (Supplementary Figure S4) that highlight a strong association between PASC-linked genes and the nervous system among Indian populations.

We identified 806 candidate genes shared between the severity and PASC cohorts. Among these, 30 protein-coding genes are associated with neuropsychiatric disorders (Table 2), while 23 genes were linked to cardiovascular conditions (Table 3). Interestingly, *calcium voltage-gated channel subunit alpha1 C (CACNA1C)*, *solute carrier family 8 member A1 (SLC8A1)*, *G protein-coupled receptor kinase 5* (*GRK5)*, *phosphodiesterase 4B (PDE4B)*, and *leucine-rich repeat kinase 2* (*LRRK2)* were common to both categories, suggesting overlapping molecular mechanisms in neurological and cardiac pathophysiology.

**TABLE 2:** Candidate protein-coding neuropsychiatric genes common to both the severity and PASC cohorts.

Gene	Linked disorder	Biological function
*CACNA1A*	Episodic ataxia and hemiplegic migraine	P/Q-type Ca^2+^ channel
*CACNA1C*	Timothy syndrome and mood disorders	Shared neuro–cardiac Ca^2+^ regulation
*GRIK3*	Mood and psychotic disorders	Ionotropic glutamate receptor
*GRM7*	Epilepsy and mood disorder	mGluR7 signaling
*CNTNAP2*	Epilepsy, language impairment, and ASD	Axon–glia contact, synaptic stability
*NLGN1*	ASD and cognitive deficits	Synaptic adhesion protein
*NRXN1*	Autism and epilepsy	Presynaptic adhesion
*DLG2*	Schizophrenia and ASD	Postsynaptic scaffolding
*DLGAP1*	Schizophrenia and neurodevelopmental	Postsynaptic density complex
*FOXP1*	Intellectual disability and speech delay	Transcriptional regulation of brain development
*RBFOX1*	Epilepsy and ASD	Splicing regulation in neurons
*MAP2*	Neuronal development	Cytoskeletal protein in dendrites
*SLC1A3*	Episodic ataxia and migraine	Astrocytic glutamate transporter
*SLC8A1*	Neural excitability	Na^+^/Ca^2+^ exchanger in neurons
*LRRK2*	Parkinson’s disease	Microglia activation and autophagy
*JAK1*	Immune neuropathies	Cytokine (IFN/IL-6) signaling
*CSMD2*	Schizophrenia	Complement regulation and synapse pruning
*ERBB3*	Neuropathy/myelination	Neuregulin axis and glia signaling
*NOS1*	Autonomic dysfunction	NO signaling and autonomic centers
*NOS1AP*	QT and ANS traits	Neuron–cardiac electrical coupling
*DAB1*	Neurodevelopmental delay	Reelin pathway and axon guidance
*DCC*	Agenesis of corpus callosum	Netrin receptor
*CTBP2*	Synaptic ribbon defects	Presynaptic transcriptional regulator
*APP*	Alzheimer’s disease and amyloid angiopathy	Amyloid processing and neurovascular coupling
*GRK5*	Alzheimer’s disease and tauopathy	GPCR/tau kinase role
*HTR4*	Mood and cognition	Serotonin receptor
*HTR7*	Depression and circadian rhythm	Serotonin receptor
*PDE4B*	Schizophrenia and depression	cAMP regulation and neuroinflammation
*ABCC2*	CNS drug metabolism	Transporter and pharmacogenomic relevance
*ZMYM4*	Neurodevelopmental syndrome	Chromatin regulation
*UNC5C*	Alzheimer’s disease, ASD, and synaptic remodeling	Induces apoptosis via caspase activation
*CTNNA3*	Alzheimer’s disease, ASD, bipolar disorder, and schizophrenia	Regulates axon and dendritic arborization, neuronal migration, and synaptogenesis

**TABLE 3:** Candidate protein-coding cardiovascular genes common to both the severity and PASC cohorts.

Gene	Linked disorder/trait	Biological function
*TTN*	Dilated cardiomyopathy	Sarcomeric titin; major structural determinant
*MYBPC3*	Hypertrophic cardiomyopathy	Myosin-binding protein C; HCM mutations
*MYH11*	Aortic aneurysm/dissection	Smooth muscle myosin heavy chain
*FBN1*	Marfan syndrome	ECM stability and elastic fiber integrity
*RYR2*	CPVT and arrhythmia	SR Ca^2+^ release and ventricular ectopy
*CACNA1C*	Long QT/Timothy syndrome	L-type Ca^2+^ channel and cardiac repolarization
*CACNA1B*	Channelopathy overlap	N-type Ca^2+^ channel and autonomic synapses
*CACNA2D3*	Arrhythmia risk (assoc.)	Auxiliary Ca^2+^ channel subunit
*KCNQ1*	Long QT	Cardiac repolarization (IK current)
*CAMK2D*	Arrhythmia/HF	CaMKIIδ in excitation–contraction coupling
*SLC8A1*	Arrhythmia/Ca^2+^ dysregulation	Na^+^/Ca^2+^ exchanger; cardiac relaxation
*PRKG1*	Vascular tone	cGMP-PKG signaling and vasodilation
*FLT1 (VEGFR1)*	Endothelial dysfunction	Angiogenesis and vascular permeability
*RGS5*	Hypertension and vascular remodeling	Regulator of vascular smooth muscle contractility
*PDGFD*	Atherosclerosis and coronary disease	Vascular inflammation and SMC proliferation
*ADAMTS17*	Connective tissue disorder	ECM remodeling and possible valve integrity
*GRK5*	Cardiac hypertrophy and HF	GPCR desensitization and heart failure signaling
*CHRM3*	BP regulation and ANS control	Muscarinic receptor and parasympathetic regulation
*ITPR2*	Hypertension and vascular Ca^2+^	IP3 receptor and vascular Ca^2+^ signaling
*PDE4B*	Heart failure and inflammation	cAMP metabolism and cardiac stress response
*RGS6*	Arrhythmia and ANS balance	Parasympathetic control and heart rate variability
*NOS1/NOS1AP*	ANS/QT traits	NO signaling and neuro-cardiac coupling
*ALDH2*	Alcohol-related heart risk	Ethanol metabolism and cardioprotection pathways
*LPA*	Atherosclerosis	Lipoprotein(a); thrombosis and CAD risk
*LRRK2*	Cardiac remodeling	Inflammation and myocardial stress response
*DSG2*	Arrhythmogenic right ventricular cardiomyopathy	Wnt/β-catenin and MAPK pathways, affecting cell proliferation and cardiac remodeling
*DSP*	ARVC and left-dominant arrhythmogenic cardiomyopathy (LDAC)	Modulates PKC, GSK3β, and Wnt/β-catenin signaling

Notably, 14 of the 54 highly significant genes prioritized by the COVID-19 Host Genetics Initiative (HGI) Consortium (Kanai et al., 2023) were also identified in either the severity or the PASC cohort or both, underscoring a strong concordance between our results and global COVID-19 host-genetic evidence (Table 4). *Anoctamin 1* (*ANO1)*, along with *protein kinase AMP-activated catalytic subunit alpha 2 (PRKAA2/AMPK)*, *heat shock protein family B (small) member 7 (HSPB7)*, *potassium voltage-gated channel subfamily Q member 1 (KCNQ1)*, and *von Willebrand factor (VWF)*, was unique to the PASC cohort. Furthermore, four PASC-linked candidate genes in the GPCR pathway were identified in the PASC cohort (Supplementary Table S3).

**TABLE 4:** Comparison of candidate genes identified in the severity and exploratory PASC cohorts that overlap with highly significant loci reported by the COVID-19 host genetics initiative consortium.

Gene	HGI: Sev/hosp/crit	Severity cohort	PASC cohort	Function	Primary system
*CCHCR1*	✓/✓/✓	✓	✓	Immune regulation	Immune
*IL10RB*	✓/✓/–	✓	​	Cytokine signaling	Immune
*KANSL1*	✓/–/–	✓	✓	Chromatin remodeling	Multisystem
*SFTPD*	✓/–/–	​	✓	Lung innate defense	Pulmonary
*SLC6A20*	✓/✓/✓	​	✓	SARS-CoV-2 entry-related transporter	Pulmonary/metabolic
*ADK*	✓/–/–	✓	✓	Purine metabolism	Neurological/immune
*CSMD2*	✓/–/–	✓	✓	Complement regulation	Neurological
*DAB1*	✓/–/–	✓	✓	Neuronal signaling	Neurological
*GRID1*	✓/–/–	✓	✓	Glutamatergic signaling	Neuropsychiatric
*LRRK2*	✓/–/–	✓	✓	Neuroimmune signaling	Neurological
*RGS5*	✓/–/–	✓	✓	GPCR/vascular tone	Cardiovascular
*ZMYM4*	–/✓/–	✓	✓	Transcriptional regulation	Multisystem
*IGF1*	✓/✓/–	​	✓	Growth factor signaling and tissue repair	Metabolic/cardiovascular
*PLSCR1*	✓/✓/✓	​	✓	Interferon-inducible lipid scramblase and antiviral response	Immune/antiviral

### Transcriptional profiling following SARS-CoV-2 infection

**FIGURE 5:**
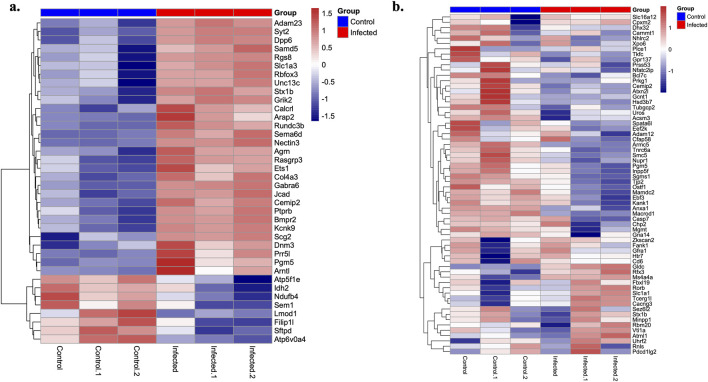
Heatmaps summarizing the differential expression pattern of candidate genes between control and SARS-CoV-2-infected hamsters in **(a)** lung and **(b)** brain tissues. These genes were also identified in the exploratory PASC cohort. Upregulation and downregulation are shown by gradients of red and blue, respectively.

Transcriptional profiling was performed using RNA sequencing (RNAseq) on brain and lung tissues obtained from SARS-CoV-2 and mock-infected hamsters. A stringent threshold (*p* < 10^−5^) was applied to reduce false positives given the exploratory sample size (N = 3 per group), which adhered to ethical principles of minimal animal use. The differential expression pattern of candidate genes between control and infected hamsters is summarized in the heatmap (Figure 5). In the lung, 115 genes were significantly downregulated (*p* < 10^−5^), and 140 genes were significantly upregulated (*p* < 10^−5^) (Supplementary Figure S5a). In the brain, 205 and 271 genes were significantly downregulated and upregulated, respectively (*p* < 10^−5^) (Supplementary Figure S5b). Among the differentially expressed candidate genes, 32 downregulated and 33 upregulated in the brain overlapped with those identified in the exploratory PASC GWAS cohort (Table 5). Similarly, 8 downregulated and 30 upregulated candidate genes in the lung were also identified in the PASC cohort (Table 6).

**TABLE 5:** Significantly upregulated and downregulated (*p* < 0.00001) candidate genes in the brains of SARS-CoV-2-infected hamsters were also identified in the exploratory PASC cohort.

Gene	Log2 fold change	*p*	Expression	Function and disease association
*Vti1a*	−6.528	1.55E-05	Downregulated	Its depletion alone has minor effects, but when ablated with *Vti1b*, it leads to perinatal lethality (Kunwar et al., 2011) and impaired neurodifferentiation (Kotschnew et al., 2024)
*Tcerg1l*	−4.527	3.18E-09	Downregulated	Enriched in the brain and retina and might regulate immunological pathways (Yi et al., 2012)
*Casp7*	−4.129	7.65E-06	Downregulated	Pro-apoptotic factor implicated in susceptibility to ischemic stroke (Zheng et al., 2019)
*Cpxm2*	−3.935	6.73E-07	Downregulated	Loci associated with left ventricular hypertrophy in rodents (Grabowski et al., 2013)
*Bcl7c*	−3.395	2.54E-07	Downregulated	Highly expressed in the brain and a significant reduction in its levels in gliomas was linked to poor prognosis (Liu et al., 2021a)
*Cd6*	−3.299	3.22E-05	Downregulated	Found in T lymphocytes and enriched in various regions of the brain (Mayer et al., 1990)
*Gcnt1*	−3.277	7.53E-13	Downregulated	Lung necrotic lesions and increased susceptibility to *Mycobacterium tuberculosis* infection (Fonseca et al., 2020)
*Plce1*	−3.126	4.81E-10	Downregulated	Locus-level pleiotropy between urate and severe COVID-19 observed on 10q23.33 containing kidney disease-related genes such as *PLCE1 46* and *NOC3L* (PMID: 36400032)
*Gldc*	−2.893	6.72E-07	Downregulated	Underlies ∼20% of all cases of glycine encephalopathy (OMIM #605899) (Huynh et al., 2023)
*Minpp1*	−2.777	8.94E-07	Downregulated	Linked with pontocerebellar hypoplasia 16 (OMIM #619527) (Ucuncu et al., 2020)
*Nupr1*	−2.737	2.92E-05	Downregulated	Regulator of iron-dependent cell death or ferroptosis that plays a role in various neurodegenerative disorders, e.g., Parkinson’s disease (Liu et al., 2021b; Costa et al., 2023)
*Armc5*	−2.629	2.06E-08	Downregulated	Poorly understood, but its depletion compromises T-cell proliferation and differentiation (Hu et al., 2017)
*Prss53*	4.464	2.11E-07	Upregulated	Susceptibility for Alzheimer’s disease in a transcriptome-wide association study (Sun et al., 2021)
*Smc5*	4.101	2.39E-06	Upregulated	Regulator of chromosome architecture and genome stability required in early brain development (Atkins et al., 2020)
*Cacng3*	3.441	4.65E-05	Upregulated	May underlie the pathogenesis of Alzheimer’s disease (AD) (Jia et al., 2021)
*Prkg1*	3.002	1.23E-06	Upregulated	Significantly associated with salt sensitivity of blood pressure (Xie et al., 2021)
*Ms4a4a*	2.741	9.60E-06	Upregulated	Expressed in microglia and associated with the risk of AD (Deming et al., 2019)
*Tjp2*	2.709	2.85E-07	Upregulated	Encodes tight junction components in the blood–brain barrier. Its mRNA levels were not altered in the brain in several neuropsychiatric disorders (Greene et al., 2020)
*Pdcd1lg2*	2.642	5.47E-08	Upregulated	Inhibits T-cell activation in physiological and pathological states (Latchman et al., 2001)
*Dhx32*	2.627	5.19E-10	Upregulated	Associated with the risk of cerebral palsy (Jin et al., 2020)
*Slc16a12*	2.498	1.84E-08	Upregulated	Highly expressed in the retina and identified in familial juvenile cataract, micro-cornea, and renal glucosuria (OMIM^*^ #612018) (Kloeckener-Gruissem et al., 2008)
*Zkscan2*	2.313	2.28E-05	Upregulated	Predicted transcription factor
*Ostf1*	2.139	1.07E-05	Upregulated	Associated with COVID-19 susceptibility and severity in the Thai population (Chamnanphon et al., 2022)
*Hsd3b7*	2.030	2.44E-07	Upregulated	Catalyzes bile synthesis (Bossi et al., 2017)
*Uhrf2*	2.026	8.22E-08	Upregulated	Regulator of DNA methylation and hydroxymethylation, along with spatial memory acquisition and retention in mice (Chen et al., 2017)

* OMIM, online Mendelian inheritance in man. It is a detailed and authoritative reference resource documenting human genes and their associated genetic traits.

**TABLE 6:** List of candidate genes that are significantly up- and downregulated (*p* < 1 × 10^−5^) in the lungs of SARS-CoV-2-infected hamsters and also identified in the PASC cohort.

Gene	Log_2_ fold change	*p*	Expression	Any known association
*Gabra6*	7.713	2.90E-12	Upregulated	Anxiety (Gonda et al., 2017)
*Rgs8*	4.604	2.48E-12	Upregulated	Regulator of mGluR1 signaling in Purkinje cells and pathology of spinocerebellar ataxias (Wu and Kapfhammer, 2021)
*Kcnk9*	3.265	2.66E-09	Upregulated	Related to Birk-Barel syndrome (OMIM #612292) with features such as intellectual disability^*^
*Grik2*	2.627	7.41E-06	Upregulated	Non-syndromic neurodevelopmental disorder with impaired language and ataxia (OMIM #619580) (Stolz et al., 2021)
*Unc13c*	2.433	5.72E-06	Upregulated	Associated with AD (Miller et al., 2013)
*Syt2*	2.119	9.09E-10	Upregulated	Associated with presynaptic distal motor neuropathy (Herrmann et al., 2014)
*Samd5*	1.952	7.03E-06	Upregulated	Retinitis pigmentosa and chondroid chordoma (OMIM #620517)^*^
*Atp6v0a4*	−1.974	3.24E-09	Downregulated	Distal renal tubular acidosis 3, with or without sensorineural hearing loss (OMIM #605239)^*^
*Grm3*	2.307	9.51E-06	Upregulated	Regulates cognition and risk of schizophrenia (Egan et al., 2004)
*Fmo5*	1.563	3.52E-05	Upregulated	Associated with sporadic amyotrophic lateral sclerosis in female individuals (Gagliardi et al., 2016)
*Pmel*	−3.329	2.17E-05	Downregulated	Associated with vitiligo-associated multiple autoimmune disease susceptibility 1 (Kemp et al., 1998)
*Arntl*	1.373	6.45E-06	Upregulated	Associated with myocardial infarction (Škrlec et al., 2020) and multiple sclerosis (Lavtar et al., 2018)
	2.465	9.30E-14	Upregulated	
*Slc1a3*	1.357	1.53E-05	Upregulated	Associated with episodic ataxia (OMIM #612656) (Chivukula et al., 2020)
	1.631	4.27E-07	Upregulated	

* The functions of the genes were obtained from GeneCards®: The Human Gene Database (https://www.genecards.org, last accessed on 01.01.2025).

### Population-specific variation in a genetic predisposition for PASC

**FIGURE 6:**
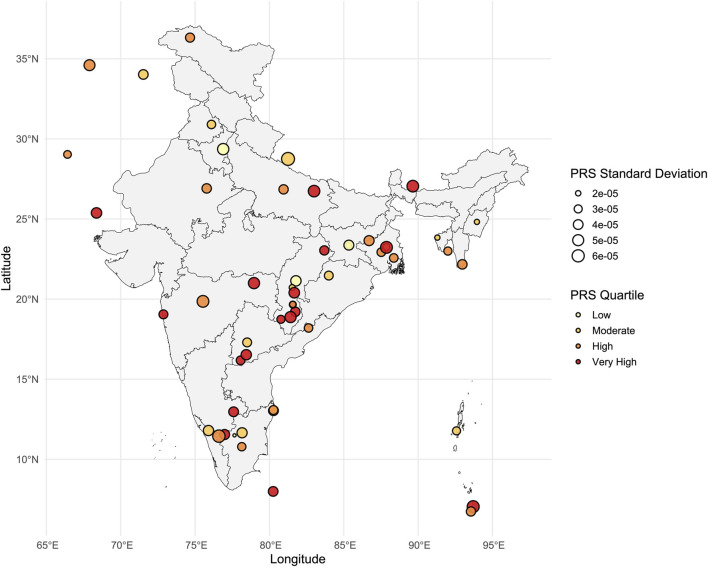
Polygenic risk score (PRS) for PASC among various South Asian populations. PRS was calculated using PRSice v2. PRS are divided into four quartiles: Q1 (low), Q2 (medium), Q3 (high), and Q4 (very high). The standard deviation (SD) of PRS is shown by the size of the circles.

The PRS map for PASC represented distinct ethnolinguistic and regional patterns, with considerable individual-specific variation indicated by the standard deviation (Figure 6). Populations such as tribal groups from Central India, Andamanese tribes, Tamilians from Sri Lanka, and Bengalis exhibited relatively elevated PRS, suggesting higher genetic propensity for PASC-related complications. In contrast, populations from North and Northwest India depicted comparatively lower polygenic risk. Populations from Southern India displayed pronounced within-group variation in genetic risk for PASC, suggesting group-specific genetic vulnerability rather than broad regional trends.

## Discussion

This study represents one of the first efforts aimed to dissect the genetic architecture underlying COVID-19 severity and its long-term sequelae in Indian populations. Our framework, combining two complimentary GWASs and integrative transcriptomic analysis, revealed convergent mechanisms and biological pathways that may underlie post-viral cardiovascular and neurological outcomes. The findings suggest that host genetic factors associated with severe acute COVID-19 may contribute to long-term vulnerability to PASC. While the limited sample-size renders the PASC–GWAS exploratory, identification of similar trends across several independent layers of data supports their biological plausibility.

### Integrative view of COVID-19 severity and susceptibility to PASC

Across both GWAS models, we uncovered variants that mapped to candidate loci influencing ECM organization, vesicular trafficking, and stress-response networks, which are not only central to crucial physiological processes, namely, cell differentiation, development, and metabolism, but also pivotal for modulation of tissue remodeling, homeostasis, and immune responses post-infection (Su et al., 2024). Accordingly, rather than representing isolated effects, these observations may allude to a global dysregulation of the cellular resilience mechanism that persists following an incomplete resolution of the SARS-CoV-2 infection and precipitates into long-term pathology.

Extensive evidence supports a bidirectional molecular axis linking the brain and the heart, wherein neural, endocrine, and inflammatory signals coordinate autonomic regulation, cardiac contractility, and metabolic adaptation (Plott et al., 2024; Ajijola et al., 2025). Congruently, disruption of the neurocardiac axis by SARS-CoV-2 infection may cause neurological and cardiovascular phenotypes, among others, as observed in PASC.

### Synaptic adhesion, axon guidance, and neurodevelopment

Multiple candidate loci that emerged from both cohorts and are central to synaptic organization and neurodevelopment include *contactin-associated protein 2 (CNTNAP2)*, which belongs to the neurexin superfamily of synaptic transmembrane adhesion molecules and is highly expressed in the brain (Kleijer et al., 2015; St. George-Hyslop et al., 2022). It is involved in the organization of myelinated neurons (Gordon et al., 2014) and neural circuit assembly (Anderson et al., 2012) and is strongly associated with susceptibility to neurodevelopmental disorders (NDDs), such as specific language impairment (SLI), autism spectrum disorder (ASD), schizophrenia, and depression (Alarcón et al., 2008; Chiocchetti et al., 2015; Fang et al., 2021). *ADAM metallopeptidase with thrombospondin type 1 motif 17 (ADAMTS17)* encodes a secreted metalloprotease that is highly expressed in the brain and retina (Morales et al., 2009) and is implicated in ECM remodeling and organization, as well as in a hereditary eye disease (Hubmacher et al., 2017). *Amyloid beta precursor protein (APP)* encodes a transmembrane protein with essential roles in many neurodevelopmental processes, such as neurogenesis, neurite outgrowth, axonal guidance, and synaptogenesis, and is central to the pathogenesis of Alzheimer’s disease (AD) (Chau et al., 2023).

Other loci that emerged from both GWASs with high relevance to CNS include *WW domain containing oxidoreductase (WWOX)*, which has been associated with various complex and rare monogenic brain pathologies, including ASD, intellectual disability (ID), and attention-deficit/hyperactivity disorder (ADHD) (Sanders et al., 2011; Harich et al., 2020), along with AD (Kunkle et al., 2019), multiple sclerosis (MS) (Beecham et al., 2013), spinocerebellar ataxia type 12 (SCAR12; OMIM #614322), and WWOX-related epileptic encephalopathy syndrome (OMIM #616211) (Aldaz and Hussain, 2020). *Membrane-associated guanylate kinase, WW*, *and PDZ domain-containing 1 and 2 (MAGI1* and *MAGI2*) function as scaffolds for nerve growth factor signaling and the recruitment of neurotransmitter receptors, such as α-amino-3-hydroxy-5-methyl-4-isoxazolepropionic acid (AMPA)- and N-methyl-D-aspartate (NMDA)-type glutamate receptors, neuroligins, and β-dystroglycan, at glutamatergic and GABAergic synapses, respectively (Deng et al., 2006; Sassoè-Pognetto et al., 2011).

Notable neuronal candidate genes whose expressions were significantly altered in the transcriptomes from SARS-CoV-2-infected hamster brains and lungs and those also identified in the PASC cohort included *calcium voltage-gated channel auxiliary subunit gamma 3 (CACNG3*), an AMPA receptor regulator whose dysregulation in the entorhinal cortex may underlie the pathogenesis of AD (Jia et al., 2021). It has also been predicted as significantly deregulated in gene expression datasets from COVID-19, AD, and Parkinson’s disease (PD) (Shi et al., 2023). *Gamma-aminobutyric acid type A receptor subunit alpha6 (GABRA6)*, although its polymorphisms are not directly implicated in neuropsychological issues, can trigger anxiety and depression symptoms when exposed to stress (Gonda et al., 2017).

ECM-related loci that emerged from PASC GWAS include *brain abundant membrane-attached signal protein 1 (BASP1)* (Iino et al., 1999; Maekawa et al., 1999; Frey et al., 2000) and *Abl interactor 2 (ABI2)* (Grove et al., 2004) that encode modulators of the actin cytoskeleton abundant in various brain regions, such as the cerebral cortex, cerebellum, hypothalamus, and olfactory bulb, with essential roles in neurite outgrowth, dendritic spine morphology, density, neuronal migration, synaptic plasticity, and axon regeneration. Decreased BASP1 levels have also been detected in AD (Musunuri et al., 2014). This cohort also identified *leucine-rich repeat transmembrane neuronal 3* (*LRRTM3*), which encodes a trans-synaptic adhesion molecule involved in excitatory synaptic assembly, connectivity, and plasticity (Um et al., 2016; Kim et al., 2022); its variants are associated with ASD among Indians (Dutta et al., 2023) and other populations (Sousa et al., 2010). The PASC GWAS also detected candidate genes modulating myelination by regulating intercell connectivity—for example, *contactin-associated protein 1* (*CNTNAP1)* (Bhat et al., 2001; Gordon et al., 2014), *fibulin-2* (*FBLN2*) (Ghorbani et al., 2024), and *tenascin C* (*TNC*) (Xie et al., 2013; Heneka et al., 2015; Tucić et al., 2021).

### Cardiac and neuronal findings

Two loci identified through both GWASs that have known overlapping roles in both the heart and brain are as follows: *Unc-5 netrin receptor C* (*UNC5C)*, a netrin signaling receptor highly expressed in the adult CNS and heart that is involved in axon guidance and repulsion of neuronal growth cones (Kim and Ackerman, 2011; Poliak et al., 2015), and its variants are associated with an increased risk of AD (Ridge et al., 2016); *catenin alpha 3 (CTNNA3)* encodes αT-catenin, a molecular linker for desmosomal and adherens junctions in the area composite of the vertebrate heart; it is linked to genetic dilated cardiomyopathy manifesting with ventricular arrhythmia, severe heart failure, and an increased risk of sudden death (Janssens et al., 2003), and it is also associated with a higher risk of ASD (Bacchelli et al., 2014).

From the PASC-only cohort, we found several candidates with known functions in the heart and CNS: apolipoprotein B (APOB), a carrier for clinically significant lipids, such as low-density lipoprotein (LDL), which not only has a strong genetic association with susceptibility to heart disease and stroke (Richardson et al., 2021) but whose rare variants are also associated with early-onset AD (Wingo et al., 2019). A-kinase anchoring protein 9 (AKAP9) is a scaffolding protein involved in protein kinase A activation (Piggott et al., 2008), leading to tau hyperphosphorylation, which is a hallmark of AD (Liu et al., 2006). Rare variants in *AKAP9* are associated with AD (Logue et al., 2014) and cardiac abnormalities, including long QT syndrome (Chen et al., 2007; Tse et al., 2021). G protein-coupled receptor kinase 5 (GRK5), which plays conserved roles in heart development (Burkhalter et al., 2013), has also been associated with cognitive dysfunction, dementia, type 2 diabetes, chronic inflammation, and heart failure (Hendrickx et al., 2018). Lysine acetyltransferase 6A (KAT6A) is a chromatin modulator associated with a rare genetic NDD characterized by brain, cardiac, and ocular abnormalities, along with growth retardation and ID (Urreizti et al., 2020; Jiang et al., 2021).

### Cardiac development

Candidates from the PASC cohort implicated in heart development and function include *ryanodine receptor 2 (RYR2)* (also identified in the severity cohort) and potassium voltage-gated channel subfamily Q member 1 (*KCNQ1)*, encoding calcium (Ca^++^) and potassium KvLQT1 (Kv7.1) channels, respectively, and controlling cardiac muscle excitation–contraction coupling. *RYR2* pathogenic mutations are associated with rare genetic arrhythmias that can cause sudden cardiac death (Lehnart et al., 2004). Several *RYR2* variants also predispose to Ca^++^ leakage, particularly under conditions of elevated catecholaminergic activity—for example, exercise and emotional stress that, in turn, may result in fatal cardiac arrhythmias (Lehnart et al., 2004; Fowler and Zissimopoulos, 2022). *KCNQ1* is one of the most prominent causes of long QT syndrome, leading to an increased risk of sudden and fatal cardiac arrest among young individuals (Goldenberg and Moss, 2008). Other genes include *RNA-binding motif protein 20* (*RBM20)*, which controls the expression of genes modulating Ca^++^ and other ion levels, such as *RYR2* (Maatz et al., 2014)*.* Variants in *RBM20* are associated with dilated cardiomyopathy that presents with an increased risk of arrhythmias and heart failure at a young age (Haas et al., 2015). Other relevant cardiac loci include *desmoglein 2* (*DSG2)* (Kant et al., 2015), *desmoplakin (DSP)* (Smith and Fuchs, 1998; Gigli et al., 2019), and *titin* (*TTN)* (Herman et al., 2012; LeWinter and Granzier, 2013) identified in both severity and PASC cohorts, and *myosin heavy chain 7 (MYH7)*, identified exclusively in the PASC cohort (Hinton et al., 2010; Yotti et al., 2019; Ritter et al., 2022).

### Stress response and proteostasis

Several candidate genes from the stress-response and proteostasis machineries also emerged from the PASC exploratory cohort; these include *activating transcription factor 6 (ATF6)*, *a* central player in ER stress and the unfolded protein response (Wang et al., 2000), which is activated following stressors such as myocardial ischemia and is known to play a protective role against ischemia/reperfusion damage (Jin et al., 2017); crystallin alpha B (CRYAB), a chaperone expressed at high levels in the brain, heart, skeletal muscle, and eye lens (Basha et al., 2012) and implicated in several types of cardiomyopathies (Thorkelsson and Chin, 2024) and the pathogenesis of MS (van Noort et al., 1995); and immune-modulatory factors such as *follistatin-like 1* (*FSTL1*) (Lara-Pezzi et al., 2008; Li et al., 2011; Wang et al., 2011) and *endoplasmic reticulum aminopeptidase 1(ERAP1)* (Mattorre et al., 2022; Țiburcă et al., 2024).

### Population-specific polygenic risk and translational relevance

Beyond locus-level findings, PRS analysis suggests significant inter-population variation in the cumulative risk burden for PASC. Cardiovascular complications and related fatalities have become a prominent healthcare concern in India in the post-COVID-19 era, frequently highlighted in the public discourse, even though their systematic monitoring has been limited. Distinct allele-frequency distributions across Indian populations emphasize ancestry-linked heterogeneity in host genomic susceptibility. This variation could partly explain the observed clinical diversity in PASC manifestations and underscore the importance of including underrepresented populations in global genomics consortia. From a translational perspective, ancestry-informed PRS frameworks could serve as predictive tools that prioritize individuals at a higher risk of neuro-cardiac sequelae following viral infection. Integrating genetic data with longitudinal clinical monitoring, autonomic testing, and imaging biomarkers would accelerate the transition from discovery to precision-medicine applications in post-COVID-19 care.

## Limitations and future directions

The exploratory PASC GWAS was constrained by its sample size (N = 37), limiting statistical power and necessitating careful and conservative interpretation. The use of population controls without information on their PASC status risks misclassification, thereby attenuating the observed effect sizes. The accrual of PASC patients was limited by challenges in patient recruitment and clinical follow-up, which are common in low- and medium-income countries. Since ethical principles constrained the hamster sample size, the transcriptomic results should also be interpreted as hypothesis-generating. Furthermore, although the WHO diagnostic criteria for PASC were followed, symptom overlap with alternative clinical conditions cannot be fully excluded, which may contribute to some degree of outcome misclassification. Future studies incorporating longitudinal clinical tracking, exclusionary diagnostics, and biomarker-based classification will improve diagnostic accuracy and homogeneity of PASC cohorts.

Nevertheless, this study provides the first genomic evidence from India linking host genetic predisposition for acute COVID-19 severity to the development of PASC, particularly through mechanisms affecting the neuro-cardiac axis. The convergence of human GWAS signals with transcriptional alterations noted in SARS-CoV-2-infected hamster tissues underscores conserved biological pathways underlying the chronic post-viral pathology, thereby lending some credence to the neuro-cardiac hypothesis of PASC. These findings advocate for the urgent need to undertake broader ancestry-inclusive genomic efforts across India and in pan-Asian populations and integrative risk-modeling strategies that combine polygenic susceptibility with clinical and environmental determinants to better predict PASC trajectories. In the future, functional studies using patient-derived cellular models, such as iPSC-generated neurons and cardiomyocytes, will be required to examine how variants in key candidate genes disrupt molecular networks, drive persistent post-COVID-19 manifestations, and guide precision approaches for early intervention.

## Data Availability

The datasets presented in this study can be found in online repositories. The names of the repository/repositories and accession number(s) can be found below: Science Data Bank, at https://www.scidb.cn/en/s/qIbqIr, and NCBI with the accession number PRJNA1222984. GWAS summary statistics are available upon request. Anonymized genotype data of the 60 participants in this study are available upon approval from the institutional ethics committee, Yenepoya Ethics Committee-1.

## Glossary

**ABCC2**: ATP-binding cassette subfamily C member 2
**ADAMTS17**: A disintegrin and metalloproteinase with thrombospondin motifs 17
**ADGRL2**: Adhesion G protein-coupled receptor L2
**ADK**: Adenosine kinase
ALDH2: Aldehyde dehydrogenase 2
**ANO1**: Anoctamin-1 (Ca^2+^-activated chloride channel)
**APP**: Amyloid precursor protein
ARHGAP32: Rho GTPase-activating protein 32
**BASP1**: Brain abundant membrane-attached signal protein 1
**CACNA1A**: Calcium voltage-gated channel subunit α1 A
**CACNA1B**: Calcium voltage-gated channel subunit α1 B
**CACNA1C**: Calcium voltage-gated channel subunit α1 C
**CACNA2D3**: Calcium voltage-gated channel auxiliary subunit α2δ3
**CAMK2D**: Calcium/calmodulin-dependent protein kinase II delta
**CCHCR1**: Coiled-coil alpha-helical rod protein 1
**CHRM3**: Cholinergic receptor muscarinic 3
**CNTNAP2**: Contactin-associated protein-like 2
**CNTNAP5**: Contactin-associated protein-like 5
**COL5A2**: Collagen type V alpha 2 chain
**COL6A2**: Collagen type VI alpha 2 chain
**CSMD2**: CUB and Sushi multiple domains 2
**CTBP2**: C-terminal binding protein 2
**CTNNA3**: Catenin alpha 3
**CXADR**: Coxsackievirus and adenovirus receptor
**DAB1**: Disabled homolog 1
**DCC**: Deleted in colorectal carcinoma (netrin receptor)
**DLG2**: Discs large MAGUK scaffold protein 2
**DLGAP1**: Discs large-associated protein 1
**DSP**: Desmoplakin
**DSG2**: Desmoglein 2
**DPP10**: Dipeptidyl peptidase-like 10
**ERBB3**: Erb-b2 receptor tyrosine kinase 3
**FBN1**: Fibrillin 1
**FLT1**: Fms-related receptor tyrosine kinase 1 (VEGFR1)
**FOXP1**: Forkhead box P1
**GATA3**: GATA-binding protein 3
**GNAO1**: G protein subunit alpha O1
**GRID1**: Glutamate ionotropic receptor delta type subunit 1
**GRIK3**: Glutamate ionotropic receptor kainate type subunit 3
**GRK5**: G protein-coupled receptor kinase 5
**GRM7**: Glutamate metabotropic receptor 7
**HSPB7**: Heat shock protein family B member 7
**HTR4**: 5-hydroxytryptamine receptor 4
**HTR7**: 5-hydroxytryptamine receptor 7
**IGF1**: Insulin-like growth factor 1
**IL-10RB**: Interleukin 10 receptor subunit beta
**ITPR2**: Inositol 1,4,5-trisphosphate receptor type 2
**JAK1**: Janus kinase 1
**KANSL1**: KAT8 regulatory NSL complex subunit 1
**KCNQ1**: Potassium voltage-gated channel subfamily Q member 1
**LPA**: Lipoprotein(a)
**LRRK2**: Leucine-rich repeat kinase 2
**MAP2**: Microtubule-associated protein 2
**MYBPC3**: Myosin-binding protein C, cardiac type
**MYH11**: Myosin heavy chain 11
**NLGN1**: Neuroligin 1
**NOS1**: Nitric oxide synthase 1
**NOS1AP**: Nitric oxide synthase 1 adaptor protein
**NPAS2**: Neuronal PAS domain protein 2
**NRXN1**: Neurexin 1
**NUP133**: Nucleoporin 133
**PDE4B**: Phosphodiesterase 4B
**PDGFD**: Platelet-derived growth factor D
**PLPP3**: Phospholipid phosphatase 3
**PLSCR1**: Phospholipid scramblase 1
**PRKAA2**: Protein kinase AMP-activated catalytic subunit alpha 2
**PRKG1**: Protein kinase, cGMP-dependent, type I
**RBFOX1**: RNA binding fox-1 homolog 1
**RGS5**: Regulator of G-protein signaling 5
**RGS6**: Regulator of G-protein signaling 6
**RUNX3**: Runt-related transcription factor 3
**RYR2**: Ryanodine receptor 2
**SFTPD**: Surfactant protein D
**SLC1A3**: Solute carrier family 1 member 3
**SLC6A20**: Solute carrier family 6 member 20
**SLC8A1**: Solute carrier family 8 member 1
**SLC2A9**: Solute carrier family 2 member 9
**TTN**: Titin
**TRPM8**: Transient receptor potential cation channel subfamily M member 8
**UNC5C**: Uncoordinated-5 homolog C
**VWF**: Von Willebrand factor
**WWOX**: WW domain-containing oxidoreductase
**ZMYM4**: Zinc finger MYM-type containing 4
