# Supplementary material for: Genomic and ancestral variations linked to the development of post-acute sequelae of SARS-CoV-2 infection in Indian populations
Source: Front Genet. 2026 Jan 6;16:1696764. doi: 10.3389/fgene.2025.1696764 (PMC12815448; doi:10.3389/fgene.2025.1696764)
Supplement: Supplementary file 1 [file DataSheet1.docx]

**Supplemental information**


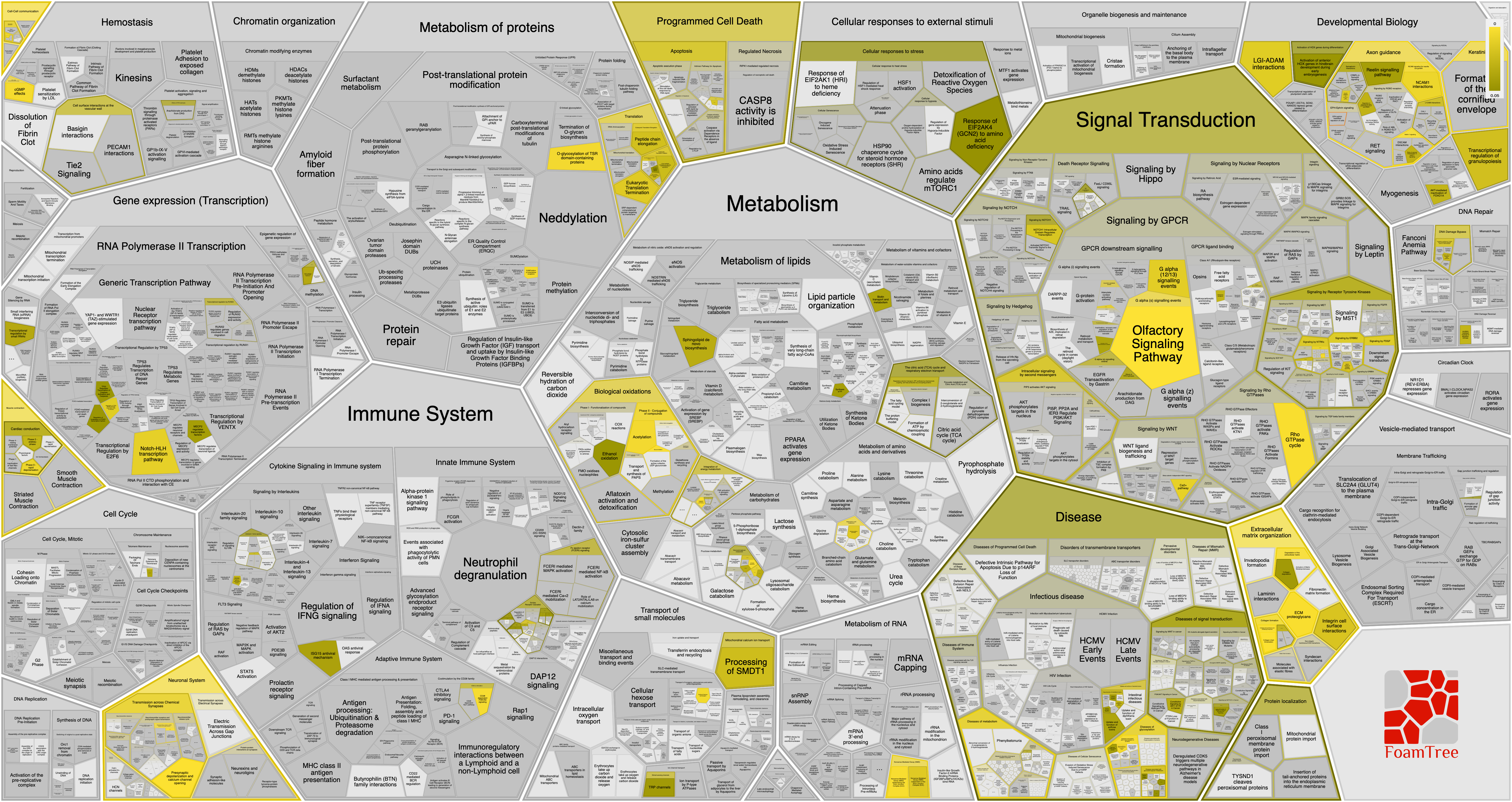


**Figure S1.** FoamTree representing various Reactome pathways associated with the significant SNPs identified in severity of infection. Pathway map was generated using SNPnexus web-based server. Pathways associated with the submitted dataset are highlighted in various shades of yellow. The gray entities represent the pathways that are represented in the query dataset but absent in the submitted dataset

**Figure S2.** Lolipop plot showing top ten GO terms (Biological Processes), the genes identified in the severity cohort are enriched with. The Bar plot was obtained through ShinyGO v0.80 web-based server. FDR was kept at 0.01


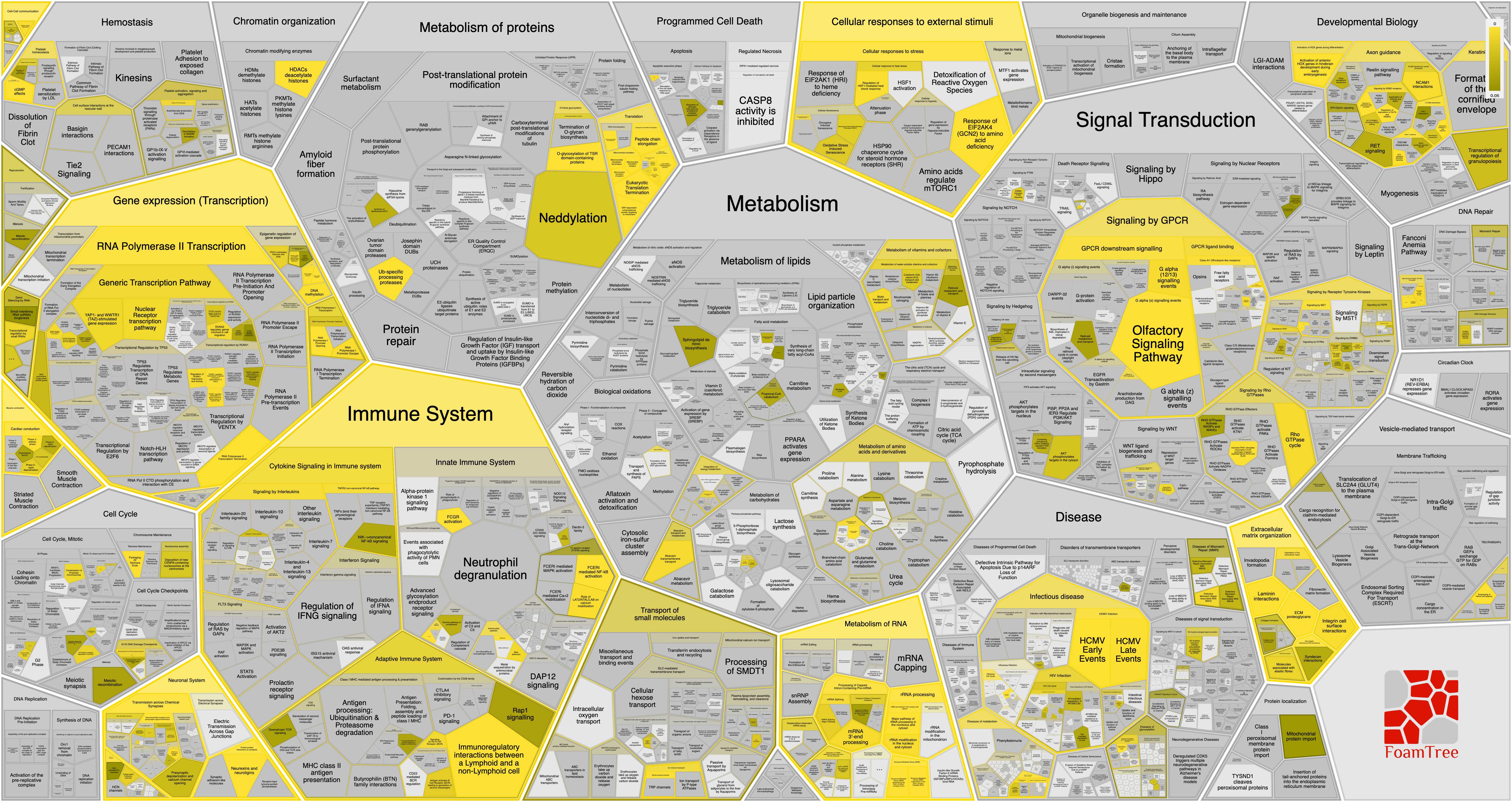


**Figure S3.** FoamTree representing various Reactome pathways associated with the significant SNPs identified in PASC. Pathway map was generated using SNPnexus web-based server. Pathways associated with the submitted dataset are highlighted in various shades of yellow. The gray entities represent the pathways that are represented in the query dataset but absent in the submitted dataset

**Figure S4.** Lolipop plot showing top ten GO terms (Biological Processes), the genes identified in the PASC cohort are enriched with. The Bar plot was obtained through ShinyGO v0.80 web-based server. FDR was kept at 0.01

**
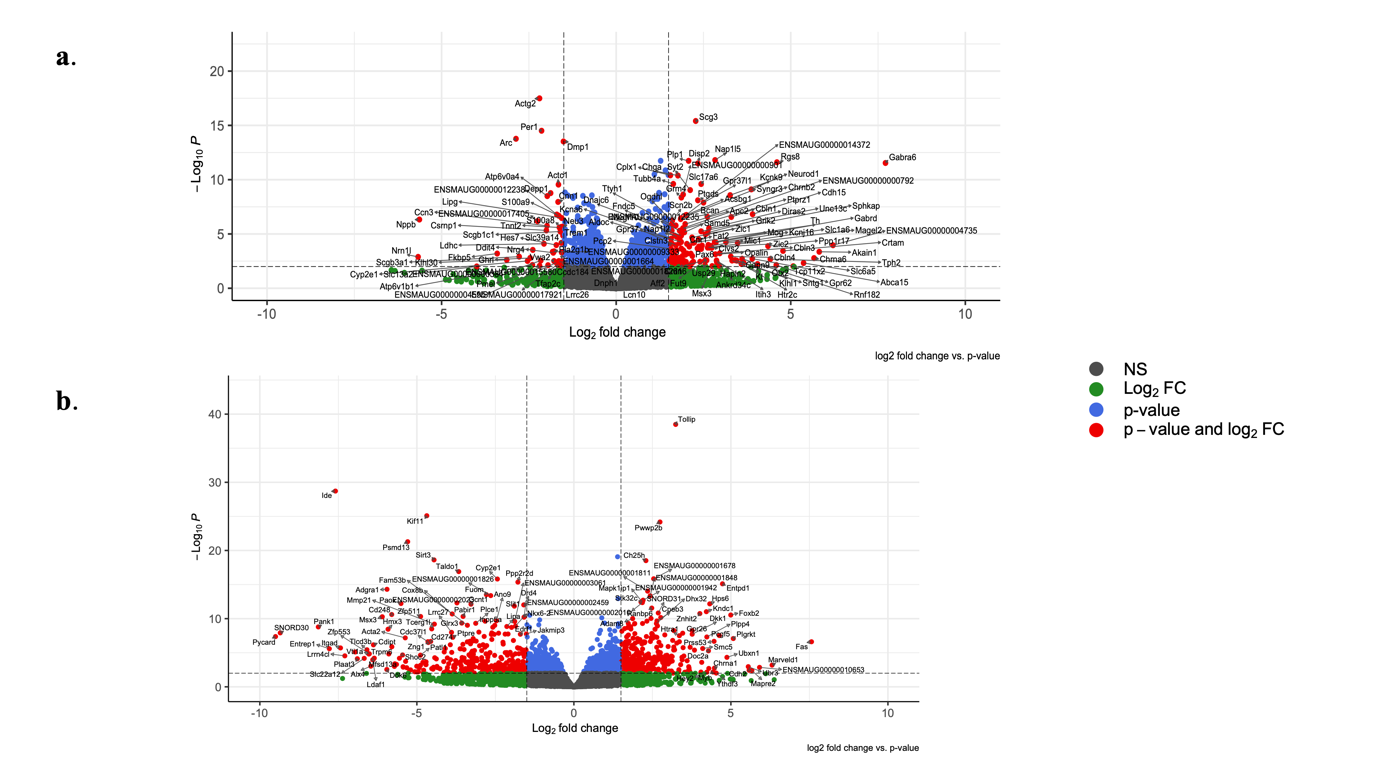
**

**Figure S5.** Transcriptomic data analysis of lung and brain tissues from young and old hamsters. Volcano plot for differentially expressed genes in a) lungs and b) brain of the hamsters. The red dots on the far right indicate genes that are significantly upregulated in the young lung tissue. The red dots on the far left indicate genes that are significantly downregulated in the old lung tissue. Black dots represent non-significant genes. The green dots indicate genes that show a substantial change in expression, but they are not statistically significant. The blue dots indicate genes that are statistically significant but do not show a substantial change in expression

**Table S1.** Symptoms considered to categorize patients into different categories

| **Asymptomatic** | **Mild** | **Moderate** | **Severe** |
| --- | --- | --- | --- |
| Individuals experiencing no noticeable or perceivable symptoms | Sore Throat, Dry Caugh, Cough producing phlegm, Runny Nose, Nasal Congestion, Fever, Chill, Headache, Body ache, Loss of Smell, Loss of Taste, Fatigue, Nausea-Vomiting, Diarrhoea, Abdominal pain | Shortness of Breath, Difficulty breathing, Mild pneumonia | Severe pneumonia, Cardiovascular problems, Other organ failure |

**Table S2.** WHO defined PASC symptoms, used to determine the PASC status of the participants

| **Symptom Domain** | **WHO-recognized Persistent Symptoms** |
| --- | --- |
| **General** | Fatigue, post-exertional malaise, fever, weight changes |
| **Respiratory & Chest** | Dyspnea (shortness of breath), chronic cough, chest pain, reduced exercise tolerance |
| **Neurological & Cognitive** | Brain fog, memory impairment, headache, dizziness, loss or distortion of smell (anosmia/parosmia), loss or distortion of taste (ageusia/dysgeusia), sleep disturbances |
| **Psychological / Mental Health** | Anxiety, depression, mood changes, post-traumatic stress, irritability |
| **Cardiovascular** | Palpitations, arrhythmia, chest tightness, myocarditis-like symptoms, postural tachycardia (POTS) |
| **Gastrointestinal** | Abdominal pain, nausea, vomiting, diarrhea, loss of appetite |
| **Musculoskeletal** | Myalgia (muscle pain), arthralgia (joint pain), muscle weakness |
| **Endocrine / Metabolic** | New-onset or worsened diabetes, thyroid dysfunction |
| **Dermatologic** | Hair loss (telogen effluvium), skin rashes |
| **Reproductive / Urogenital** | Menstrual irregularities, erectile dysfunction, urinary discomfort |
| **Ear, Nose & Throat (ENT)** | Sore throat, tinnitus, ear pain, sinus symptoms |
| **Autonomic & Systemic Dysregulation** | Orthostatic intolerance, dysautonomia, temperature instability |

**Table S3.** Candidate genes in GPCR pathways identified in this study

| **Gene** | **Function / Role** | **Cohort Identified** | **Key Notes** |
| --- | --- | --- | --- |
| *ADGRL2* | Adhesion G-protein-coupled receptor; synaptic organization and neuronal signaling | PASC and Severity | Implicated in neurodevelopment and synaptic GPCR signaling |
| *CHRM3* | Muscarinic acetylcholine receptor M3; parasympathetic and vascular smooth muscle GPCR | PASC and Severity | Regulates vasodilation, airway tone, and cardiac function |
| *GNAO1* | G-protein alpha O subunit; GPCR transducer in neuronal and cardiac pathways | PASC and Severity | Modulates neurotransmission and autonomic tone |
| *GRK5* | G-protein receptor kinase 5; phosphorylates and desensitizes GPCRs | PASC only | Cardiac remodeling, β-adrenergic receptor regulation |
